# Collagen density promotes mammary tumor initiation and progression

**DOI:** 10.1186/1741-7015-6-11

**Published:** 2008-04-28

**Authors:** Paolo P Provenzano, David R Inman, Kevin W Eliceiri, Justin G Knittel, Long Yan, Curtis T Rueden, John G White, Patricia J Keely

**Affiliations:** 1 Department of Pharmacology, University of Wisconsin, Madison, WI 53706, USA; 2 Molecular Biology Program, Laboratory for Optical and Computational Instrumentation, University of Wisconsin, Madison, WI 53706, USA; 3 University of Wisconsin Paul P. Carbone Comprehensive Cancer Center, University of Wisconsin, Madison, WI 53706, USA

## Abstract

**Background:**

Mammographically dense breast tissue is one of the greatest risk factors for developing breast carcinoma. Despite the strong clinical correlation, breast density has not been causally linked to tumorigenesis, largely because no animal model has existed for studying breast tissue density. Importantly, regions of high breast density are associated with increased stromal collagen. Thus, the influence of the extracellular matrix on breast carcinoma development and the underlying molecular mechanisms are not understood.

**Methods:**

To study the effects of collagen density on mammary tumor formation and progression, we utilized a bi-transgenic tumor model with increased stromal collagen in mouse mammary tissue. Imaging of the tumors and tumor-stromal interface in live tumor tissue was performed with multiphoton laser-scanning microscopy to generate multiphoton excitation and spectrally resolved fluorescent lifetimes of endogenous fluorophores. Second harmonic generation was utilized to image stromal collagen.

**Results:**

Herein we demonstrate that increased stromal collagen in mouse mammary tissue significantly increases tumor formation approximately three-fold (*p *< 0.00001) and results in a significantly more invasive phenotype with approximately three times more lung metastasis (*p *< 0.05). Furthermore, the increased invasive phenotype of tumor cells that arose within collagen-dense mammary tissues remains after tumor explants are cultured within reconstituted three-dimensional collagen gels. To better understand this behavior we imaged live tumors using nonlinear optical imaging approaches to demonstrate that local invasion is facilitated by stromal collagen re-organization and that this behavior is significantly increased in collagen-dense tissues. In addition, using multiphoton fluorescence and spectral lifetime imaging we identify a metabolic signature for flavin adenine dinucleotide, with increased fluorescent intensity and lifetime, in invading metastatic cells.

**Conclusion:**

This study provides the first data causally linking increased stromal collagen to mammary tumor formation and metastasis, and demonstrates that fundamental differences arise and persist in epithelial tumor cells that progressed within collagen-dense microenvironments. Furthermore, the imaging techniques and signature identified in this work may provide useful diagnostic tools to rapidly assess fresh tissue biopsies.

## Background

Mammographically dense breast tissue is linked to a greater than four-fold increased risk of breast carcinoma [1-3], and is one of the greatest independent risk factors for breast cancer [1,2,4]. For instance, breast density in more than 50% of the tissue may account for up to 30% of breast cancers, while BRCA1 and BRCA2 mutations, although conferring a greater relative risk, account for only 5% of breast cancers (see Boyd et al [5] and references therein). Breast tissue density is additionally increased with hormone replacement therapy [6], suggesting increased density may be part of the underlying mechanism by which hormone replacement therapy increases cancer risk. Furthermore, ductal carcinoma *in situ *(DCIS), a local precursor to some invasive breast cancers, arises overwhelmingly in dense regions of the breast [7]; and high breast tissue density is associated with a shift to more malignant tumors [8], an increased likelihood of DCIS [8,9], invasive breast carcinoma [9,10], lymphatic and vascular invasion [11], and an approximately three-fold greater risk of developing a second breast carcinoma [10]. However, although there is considerable correlative data identifying breast density as a risk factor for developing carcinoma, a causal link between breast density and carcinoma has not been established. Moreover, the molecular mechanisms driving breast density-related tumor formation and progression remain largely unknown.

Importantly, areas of increased breast density are not only associated with increased epithelial and stromal cellularity [12-14], but also significantly increased fibrillar collagen deposition [8,13,14]. In addition, it has been reported that levels of total collagen increase as radiographic breast tissue density increases [8,13]. This is significant since tissue microenvironments play an important role in maintaining normal cellular behavior [15,16], and stroma surrounding breast epithelial cells is believed to be critically involved in epithelial transformation, carcinoma growth, and metastasis [17-21]. Consistent with this concept, adipose-derived type VI collagen promotes tumor growth [22], while disturbing the epithelial-stromal interaction by disrupting the β1-integrin in mammary epithelial cells inhibits tumorigenesis [23]. A less considered aspect of the complexity of the epithelial-stromal interaction is the fact that the stroma is a dynamic mechanical microenvironment, with dense collagenous stroma transmitting multi-axial deformations to breast cells during tissue deformation and increasing resistance to cellular contractility. Such mechanical signals arising from increased density or rigidity of the microenvironment play a role in the transformed phenotype of breast epithelial cells [24,25]. Hence, although tumor formation is a multistep process involving genetic alterations of the epithelial cell, it has become clear that the epithelial-stromal interaction plays a crucial role in tumor formation and progression. Therefore, due to the increased stroma associated with breast tissue density we hypothesized that increasing collagen density in the mammary gland would promote tumorigenesis. Although there is a strong correlative link between breast density and carcinoma, to date collagen density has not been causally linked to tumorigenesis, largely because studies utilizing animal models with different stromal density have not been performed previously. Here we demonstrate that mammary tumor formation, invasion, and metastasis are enhanced in collagen-dense stroma in a transgenic mouse model.

## Methods

### Mice

The University of Wisconsin animal use and care committee approved this study. Breeding pairs of Col1a1^tmJae ^mice [26] in the B6/129 background were obtained from Jackson Laboratory. Male FVB Polyomavirus middle-T mice under the control of the mammary specific MMTV promoter were originally obtained from Dr Amy Moser (University of Wisconsin) and are abbreviated PyVT following the Jackson Laboratory (from which they originated) nomenclature, but are also commonly abbreviated as PyMT or PyV MT. Col1a1^tmJae ^homozygote males were crossed to C57BL/6 females to generate heterozygous females that were crossed to PyVT males to generate mice with normal and collagen-dense mammary tissues carrying the polyoma transgene. Mice were palpated every 2 to 3 days starting at 8 weeks of age to identify tumors. Genotyping by polymerase chain reaction (PCR) was performed on DNA extracted from tail biopsies (Wizard SV Genomic DNA Purification System, Promega, Madison, WI) using primers indicated in the strain information provided by The Jackson Laboratory. Mice were examined for palpable tumors starting at eight weeks of age and humanely killed at 15 weeks or when the tumor burden became excessive.

### Histology and mammary gland whole mounts

Selected mammary tissues and tumors were fixed in 4% paraformaldehyde in phosphate buffered saline (PBS) and then embedded in paraffin. In addition, all tissues imaged with multiphoton microscopy were subsequently fixed and processed for histology. Tissue sections were stained with hematoxylin and eosin (H&E) with adjacent sections stained with the selective collagen stain, picrosirius red. Mammary whole mounts were prepared by fixing tissues in Carnoy's solution (10% glacial acetic acid, 30% chloroform, and 60% absolute ethanol), followed by rehydration and staining with carmine alum. Tissues were then dehydrated, cleared with xylene, and mounted.

### Immunofluorescence

Immunofluorescent staining of mammary epithelial cells was performed in a manner similar to the methodology described by Wozniak and co-workers [25]. Briefly, collagen gels were fixed in 4% paraformaldehyde for 20 minutes at room temperature. Following three washes in PBS, paraformaldehyde fluorescence was quenched with 0.15 M glycine in PBS then the gels were washed with PBS. Triton-X (0.2%) was added to permeabalize the cells, and gels blocked overnight with 2.5% fatty acid-free bovine serum albumin (BSA) + 1% donkey serum. Cell proliferation was then examined by staining with the anti-Ki-67 (mouse clone 7B11; Zymed) primary antibody in PBS containing 1% donkey serum for 30 minutes at room temperature. Following five 10-minute washes in PBS, gels were incubated with anti-mouse tetramethylrhodamine isothiocyanate (TRITC; Jackson ImmunoResearch Laboratories) secondary antibody, phalloidin-fluorescein isothiocyanate (FITC; Jackson ImmunoResearch Laboratories), and bisbenzimide (Sigma-Aldrich) in PBS containing 1% donkey serum for 30 minutes at room temperature. Gels were again washed with PBS and mounted with Prolong Antifade mounting media (Molecular Probes). Imaging was performed on a TE300 Nikon epifluorescence inverted microscope with acquired z-stacks deconvolved using Slidebook imaging software (Olympus).

### Lung metastasis

Lungs from PyVT/wt (*n *= 4) and PyVT/Col1a1 (*n *= 4) mice (as well as wt/wt and wt/Col1a1 as negative controls) were harvested at 15 weeks, fixed in formalin, and processed for histology. Sections were cut every 50 μm through the entire tissue and sections stained with H&E. Total lung metastases over all sections were then counted.

### Three-dimensional invasion assay

Uniform sized tumor explants were harvested from intact tumors using a tissue biopsy punch (3 mm diameter), rinsed with PBS (containing 100 units penicillin, 100 μg streptomycin, and 0.25 μg/ml amphotericin B), and placed into 2.0 mg/ml collagen gels (BD Biosciences, San Diego, CA) that were neutralized with 2× HEPES buffer. Tumors were maintained in collagen gels floated in Dulbecco's Modified Eagle's Medium (DMEM) containing 5% fetal bovine serum (FBS), penicillin (100 units), streptomycin (100 μg), and amphotericin B (0.25 μg/ml) for 10 days over which time the number of distant multicellular colonies were counted.

### Isolation of tumor cells and migration assay

Tumors from PyVT/wt and PyVT/Col1a1 backgrounds were minced and digested with 2 mg/ml collagenase and 10 μg/ml hyaluronidase in DMEM containing penicillin (100 units), streptomycin (100 μg), and amphotericin B (0.25 μg/ml). Following gentle shaking at 37°C for 3 hours, cells were pelleted, washed, and plated in DMEM containing 5% FBS. Thirty-six hours post-harvest the tumor cells were transferred to Transwell plates (Corning Inc, Corning, NY) using serum and soluble collagen containing media as the chemoattractant.

### Multiphoton laser-scanning microscopy

For live tissue imaging by multiphoton excitation (MPE) and second harmonic generation (SHG), mammary tumors were harvested and live tissue maintained in buffered media at 37°C. All tissues were imaged immediately following tissue harvest using an Optical Workstation [27] that was constructed around a Nikon Eclipse TE300. A Tsunami Ti:sapphire laser driven by a Millenia 5 W pump laser (Spectra Physics, Mountain View, CA) excitation source producing around 100 fs pulse widths and tuned to 890 nm was utilized to generate both MPE and SHG. The beam was focused onto the sample with a Nikon 60X Plan Apo water-immersion lens (numerical aperture (NA) = 1.2). All SHG imaging was detected from the back-scattered SHG signal [28], and the presence of collagen confirmed in our tissues using fluorescence lifetime imaging microscopy (FLIM) on the same optical workstation, since the SHG from collagen has no lifetime. Furthermore, owing to the fundamentally different physical behavior of MPE and SHG, signals could be discriminated by filtering the emission signal. We used a 464 nm (cut-on) long pass filter to isolate the emission from autofluorescence from the conserved 445 nm SHG emission. A 445 nm (narrow-band pass) filter was therefore used to isolate the SHG emission. Acquisition was performed with WiscScan [29] a software acquisition package developed at LOCI (Laboratory for Optical and Computational Instrumentation, University of Wisconsin, Madison, WI) and image analysis for MPE/SHG was performed with ImageJ and VisBio [30] software. For TACS-1 image analysis, additional surface rendering plug-ins for ImageJ were utilized [31]. For TACS-2 and -3, ImageJ was used to quantify the collagen fiber angle relative to the tumor. The tumor boundary was defined and the angle relative to the tangent of tumor boundary was measured every 10 μm as reported previously [27].

### Fluorescence and spectral lifetime imaging microscopy

FLIM was performed on live tissue with the optical workstation described above and as described previously [27]. Briefly, the Ti:sapphire laser (Millennium/Tsunami, Spectra-Physics, Mountain View, CA) was tuned to 890 nm with the beam focused onto the sample with a Nikon 60X Plan Apo water-immersion lens (NA = 1.2). Intensity and FLIM data were collected by a H7422 GaAsP photon-counting photomultiplier tube (PMT; Hamamatsu, Bridgewater, NJ) connected to a time-correlated single photon counting (TCSPC) system (SPC-730, Becker & Hickl, Berlin, Germany). Multiphoton spectral lifetime imaging microscopy (SLIM) was performed using a second-generation system that evolved from a previously described instrument [32] built around an inverted microscope (Eclipse TE2000, Nikon, Melville, NY). Briefly, a Mira Ti:sapphire mode-locking laser driven by a Verdi 8 W laser (Coherent Mira, Coherent, Santa Clara, CA) was used to generate pulse widths of approximately 120 fs at a repetition rate of 76 MHz. Intensity and fluorescence lifetime data were collected over 16 individual 10 nm spectral-width channels using a 16-anode photon counting linear PMT array (PML-16, Becker & Hickl) connected to a TCSPC system (SPC-830, Becker&Hickl). Fluorescent lifetime analysis from FLIM and SLIM was carried out with SPCImage (Becker & Hickl) as well as with a LOCI created computational tool, SlimPlotter [33], which allows visualization and analysis of the lifetimes by spectral channel.

### Statistical analysis

For multi-group comparisons, one-way analysis of variance (ANOVA) with a *post-hoc *Tukey-Kramer test was used. We performed *t*-testing for two-group comparisons.

## Results

### Increased collagen density promotes mammary epithelial cell proliferation in reconstituted three-dimensional matrices

**Figure 1:**
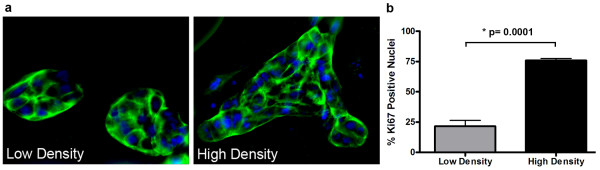
**Increased collagen matrix density directly promotes epithelial cell proliferation**. **(a) **Actin staining to visualize MCF10A human mammary epithelial cells cultured within low (1.3 mg/ml) and high-density (3.0 mg/ml) collagen gels for 21 days (actin, green; nuclei, blue). Left: Two well-differentiated acini structures formed in low-density matrices. Right: A single, less-organized colony. **(b) **Increased proliferation of mammary epithelial cells cultured within high-density matrices, measured by increased detection of the Ki67 antigen, a marker of proliferation.

To test the hypothesis that increased collagen density can directly promote growth of mammary epithelial cells in the absence of stromal cells, human MCF10A cells were cultured within three-dimensional collagen gels and proliferation was measured (Figure 1). Cells cultured within low-density matrices form well-differentiated acini structures, while colonies that formed within high-density matrices are larger, less-organized structures (Figure 1a). In agreement with the formation of larger colonies, proliferation of human mammary epithelial cells is increased in high-density matrices (Figure 1b), indicating that increasing collagen matrix density can directly promote epithelial cell proliferation.

### Increased tumor incidence in collagen-dense mammary tissues

**Figure 2:**
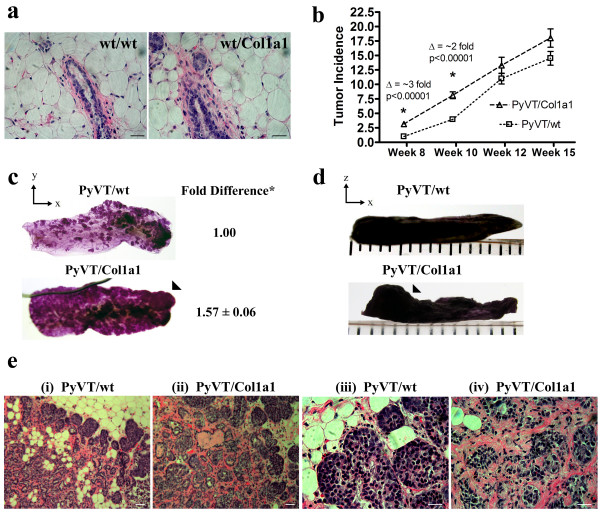
**High mammary collagen density promotes tumor formation**. **(a) **Histology of mammary glands from 10-week-old wild-type and heterozygous Col1a1^tmJae ^mice showing increased stromal collagen and hypercellularity associated with the Col1a1^tmJae ^mouse model. Scale bar 25 μm. **(b) **Significantly increased tumor numbers per mouse in collagen-dense (Col1a1) mammary glands. **(c) **Whole mount preparations of the fourth inguinal mammary glands from PyVT/wt and PyVT/Col1a1 mice at 10 weeks of age. Quantitative analysis of the area of hyperplasia from three pairs of glands calculated from a common threshold value set with density slicing in ImageJ software revealed a greater than 1.5-fold increase in hyperplasia associated with increased stromal collagen (*t*-test: *p *= 0.03). In addition, at age-matched time points, tumors in mice with dense stroma not only displayed more hyperplastic area but also tumor regions that grew out away from the gland (arrows in (c) and (d)). **(e) **Low (*i*), (*ii*) and high (*iii*), (*iv*) magnification images of H&E stained histology sections from 10-week-old mice showing increased collagen in PyVT/Col1a1 tumors ((*ii*) and (*iv*)) and a more invasive phenotype when compared with PyVT/wt (*i*) and (*iii*) tumors. Scale bars 50 μm in (*i*) and (*ii*) and 25 μm in (*iii*) and (*iv*).

In order to develop a murine tumor model possessing collagen-dense mammary tissue, we examined the mammary tissues from Col1a1^tmJae ^transgenic mice (Figure 2a). These mice carry mutations near the highly conserved matrix metalloproteinase (MMP) cleavage site for type I collagen (between Gly_775 _and Ile_776 _of the α1(I) chain) that make the collagen resistant to human collagenase digestion [26]. Although an additional cleavage site on type I collagen is vulnerable to rodent collagenase (often termed rat collagenase) and the collagen is susceptible to other proteases [26], these are not sufficient to achieve the proper balance of collagen synthesis and degradation, resulting in excessive collagen accumulation in the skin, uterus, and bone [26]. These phenotypes raised the possibility that the mammary gland, which undergoes dynamic changes in collagen deposition and degradation during development, puberty, and estrous, would rapidly accumulate excess stromal collagen. To explore this possibility, we previously analyzed mammary glands from wild-type, heterozygous, and homozygous Col1a1^tmJae ^mice. Using techniques specific for collagen detection, we reported a greater than 2.5-fold increase in stromal collagen associated with heterozygous or homozygous mice when compared with wild-type mice [27] (Figure 2a).

With a defined model for breast tissue density in place, we set out to test the hypothesis that increased mammary collagen density increases tumor formation. Mammary tumors were initiated with the PyVT transgene. This breast tumor model correlates well with many features of human cancer, progresses from hyperplasia to adenoma to early and late carcinoma [34], and is reliably invasive and metastatic [34]. When mice carrying the PyVT transgene under the control of the mammary epithelial-specific MMTV promoter were crossed with heterozygous Col1a1^tmJae ^mice, we observed an approximately three-fold increase in early tumor formation in collagen-dense tissues (that is, a three-fold greater number of tumors per mouse; see Figure 2b). This trend of increased tumor incidence in collagen-dense glands continued through week 15 (Figure 2b), and two additional PyVT/Col1a1 mice needed to be euthanized by week 13 due to excessive tumor burden (not shown). Consistent with these observations, quantitative analysis of whole mounts of the fourth mammary gland (*n *= 3 pairs) show significantly increased areas of hyperplasia (Figure 2c) with collagen-dense tissues showing increased cell growth out from the gland (Figure 2c* arrowhead *and Figure 2d). Furthermore, tumors progressing in collagen-dense tissues at 10 weeks had a more invasive morphology (Figure 2e). Of note is the fact that tumors arising in collagen-dense mammary tissue retain increased collagen density (Figure 2e and confirmed with collagen selective picrosirius red staining (not shown)). In fact, collagen levels in PyVT/Col1a1 tumor-bearing glands appear to be increased relative to non-tumor bearing collagen-dense glands (Figure 2e). This observation possibly indicates an amplified shift in the imbalance between collagen synthesis and degradation in the Col1a1 mice following tumor initiation, and may represent an increased desmoplastic response.

### Increased invasion and metastasis associated with dense stromal collagen

**Figure 3:**
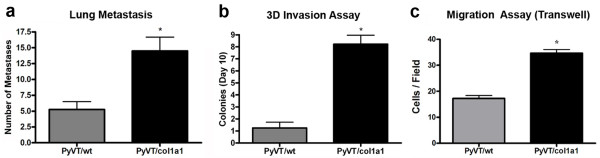
**Increased metastasis associated with dense stromal collagen**. **(a) **Increased number of lung metastases per lung at 15 weeks in mice that formed tumors in collagen-dense mammary glands (PyVT/Col1a1) when compared with mice that formed tumors in control glands (PyVT/wt; mean ± standard error of the mean (SEM), *n *= 4 of each genotype). **(b) **Three-dimensional tumor cell invasion assay showing that tumor explants from collagen-dense tumors (PyVT/Col1a1) resulted in more invasion into three-dimensional collagen gels and colony formation after 10 days than explants from PyVT/wt tumors (mean ± SEM; *n *= 4 PyVT/wt and *n *= 14 PyVT/Col1a1 tumor explants from four sibling mice). **(c) **Tumor cells extracted from collagen-dense tumors (PyVT/Col1a1) showed increased migration when compared to tumor cells from control tumors (PyVT/wt) as measured by transwell migration assays with serum as the chemoattractant (mean ± SEM; *n *≥ 3 independent experiments). *Indicates a statistically significant difference (*p *< 0.05) following analysis with *t*-tests.

Examination of later-stage tumors (week 15) demonstrated that both PyVT/wt and PyVT/Col1a1 tumors were invasive (data not shown), confirming an earlier report that late-stage wild-type PyVT tumors have invasiveness associated with collagen reorganization [27]. Moreover, since the MMTV-PyVT tumor model reliably results in lung metastases, we examined lung tissue in late-stage mice (week 15). In animals in which tumors were initiated and progressed in a collagen-dense microenvironment, a significant increase in the number of lung metastases was observed (Figure 3a). This raised the possibility that increased lung metastasis may be the result of a more-invasive and migratory cell population, or may result from the earlier onset of invasiveness. To address this question, we isolated tumor plugs and tumor cells and performed invasion and migration assays, respectively.

Data showing increased invasion in tumors that arose in collagen-dense tissue was obtained by quantifying invasion from tumor explants into three-dimensional collagen gels. To determine whether local invasion was a simple reflection of increased local collagen that facilitates invasion or also due to an intrinsic property of tumor cells arising in a collagen-dense stroma, tumor explants of defined size were placed into three-dimensional collagen gels and the number of distant colonies was counted. After 10 days in culture, explants from collagen-dense tissues resulted in significantly more colonies (Figure 3b). Furthermore, tumor cells isolated from collagen-dense tissues were in fact more migratory (Figure 3c), indicating that the earlier onset of invasiveness is likely not the sole cause for increased metastasis but that the tumor cells themselves are more invasive (Figure 3b) and migratory (Figure 3c).

### Changes in the tumor-stromal interaction associated with increased stromal collagen

**Figure 4:**
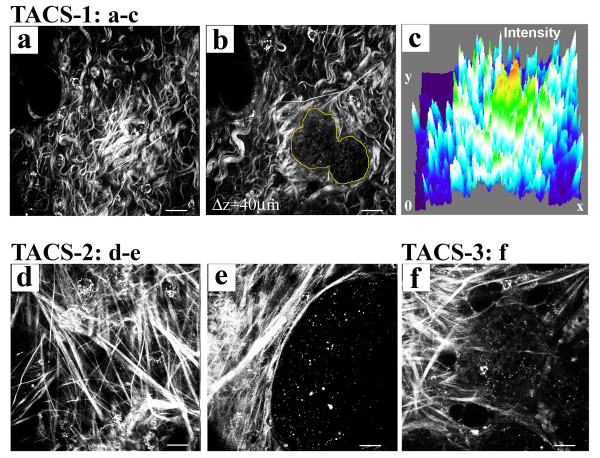
**Tumor-associated collagen signatures**. **(a)-(c) **Example of TACS-1. A region of locally dense collagen **(a) **near (40 μm 'above') a small tumor region **(b) **that is within the globally increased collagen region surrounding tumors, resulting from increased SHG (collagen) signal intensity; **(c) **three-dimensional surface plot of intensity  showing an approximately three-fold signal increase at TACS-1. **(d)**, **(e) **Example of TACS-2, showing straightened (taut) collagen fibers stretched around and constraining an expanded epithelial tumor volume. At regions of TACS-2, quantitative analysis [27] of fiber angles relative to the tumor boundary shows a distribution of fibers around 0° that correlates to non-invading regions of tumor cells. **(f) **Example of TACS-3, showing radially aligned collagen fibers, reorganized by tumor cells, at regions of tumor cell invasion. At regions of TACS-3, quantitative analysis [27] of fiber angles relative to the tumor boundary shows a distribution of fibers around 90° that correlates with local invasion of tumor cells.

Collagen content, fiber structure, and organization are potentially key determinants of tumor cell behavior [27,35]. Therefore, to better understand the reported increase in invasion and metastasis associated with increased collagen density, we employed nonlinear optical imaging of tumor-stromal interactions in intact live tumors. Multiphoton laser-scanning microscopy (MPLSM) was used to simultaneously generate intrinsic signals from cellular autofluorescence by MPE and fibrillar collagen by SHG [27,36-38]. Using this approach we previously defined [27] three Tumor-Associated Collagen Signatures (TACS; Figure 4) in mammary tumors from both Wnt-1 and PyVT transgenic mice. Specifically: TACS-1, the presence of locally dense collagen (Figure 4a) within the globally increased collagen concentration surrounding tumors, indicated by increased signal intensity (Figure 4c) at a region near the tumor, which serves as a reliable hallmark for locating small tumor regions (Figure 4b); TACS-2, straightened (taut) collagen fibers stretched around the tumor, constraining the tumor volume (Figure 4d and 4e); and TACS-3, identification of radially aligned collagen fibers that facilitate local invasion (Figure 4f). With TACS-3, a distribution of collagen fiber angles around 90° relative to the tumor boundary was indicative of high levels of local invasion while a distribution around 0° was associated with non-invading regions of the tumor [27].

**Figure 5:**
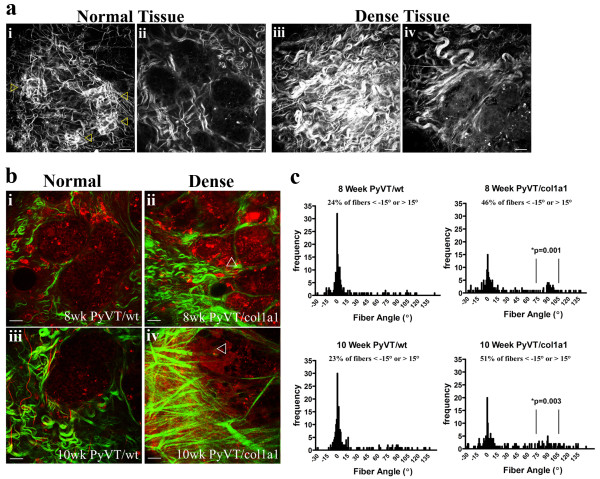
**Increased progression of tumor-associated collagen signatures and increased local invasion with high collagen density**. **(a) **TACS-1 in 8-week-old normal (wild-type; (*i*), (*ii*)) and collagen-dense (col1a1; (*iii*), (*iv*)) tumors showing more developed TACS-1 associated with density (early transition between TACS-1 and -2) while showing very early TACS-1 formation in wild-type tumors (shown with yellow arrowheads; white arrowhead indicates a TACS-1 region that is not shown since it is out of the focal plane). The displayed tumor regions ((*ii*) and (*iv*)) are at a Δ*z *= 40 μm from collagen signatures ((*i*) and (*iii*)). Note the increased endogenous cellular autofluorescence associated with tumor cells in collagen-dense tissues when PyVT/wt (*ii*) and PyVT/Col1a1 (*iv*) tumors were imaged sequentially at the same power settings ((*ii*) versus (*iv*)). Representative of *n *= 4 pairs of tumors. **(b) **Tumors were imaged and MPE (pseudo-colored red) and SHG (pseudo-colored green) signals were separated. At 8 weeks tumors showed early TACS-3 regions and some local invasion in collagen-dense tumors (*ii*) while PyVT/wt tumors (*i*) were still primarily bound by collagen (TACS-2) and non-invasive. At 10 weeks, tumors from dense tissues (*iv*) displayed further regions of TACS-3 progression and an invasive phenotype, compared to control tissues (*iii*) that were largely non-invasive and had little collagen reorganization. Representative of *n *≥ 6 tumors from each background. **(c) **Quantitative analysis of collagen fiber angles relative to the tumor boundary for 8-week (*top*) and 10-week (*bottom*) old animals. PyVT/wt animals displayed little TACS-3 and are primarily non-invasive with only 23% (8 weeks) and 24% (10 weeks) of their fibrils having angles outside of the TACS-2 distribution around 0° (that is less than -15° or more than 15°). In contrast PyVT/Col1a1 tumors were more invasive, possessing a broader fiber distribution and some regions of TACS-3 (distribution around 90°), with 46% and 51% of the fibrils distributed outside of the TACS-2 distribution (0°) at 8 weeks and 10 weeks, respectively (*indicates that the number of events associated with TACS-3/invasion (75° to 105°) was significantly greater). Calculated from at least 185 of tumor regions from at least 6 separate tumors. All scale bars are 25 μm.

In comparing tumors in the wild-type and heterozygous Col1a1^tmJae ^backgrounds carrying the MMTV-PyVT transgene, we identified critical differences in the temporal progression in density-associated tumors (Figure 5). At 8 weeks of age, TACS-1 formation in wild-type tumors (Figure 5a) was not yet well developed, and tumors were primarily non-invasive with collagen fibers distributed around 0° (Figure 5b and 5c). In contrast, collagen-dense tumors (PyVT/Col1a1) displayed more developed TACS-1 with increased collagen signal and more straightened fibers, indicating early progression to TACS-2 (Figure 5a) and some regions of TACS-3 (Figures 5b and 5c). Dense tissues (PyVT/Col1a1) began to show regions of local invasion at 8 weeks (Figure 5b-ii; highlighted with and arrowhead) corresponding to an increased frequency of reorganized collagen fibers with a peak realignment near 90° (Figure 5c). By 10 weeks of age this difference was enhanced. While tumors from PyVT/wt animals were still largely non-invasive, tumors that arose in collagen-dense tissues continued to have more collagen signal, enhanced realignment to TACS-3, and increased local invasion (Figures 5b and 5c), supporting histological findings shown in Figure 2e. Moreover, this shift in the temporal onset of TACS-3 to an earlier occurrence in collagen-dense tumors indicates the more advanced and invasive state of these tumors.

### Spectral-lifetime imaging of the tumor-stromal interaction suggests a metabolic signature associated with invasion

**Figure 6:**
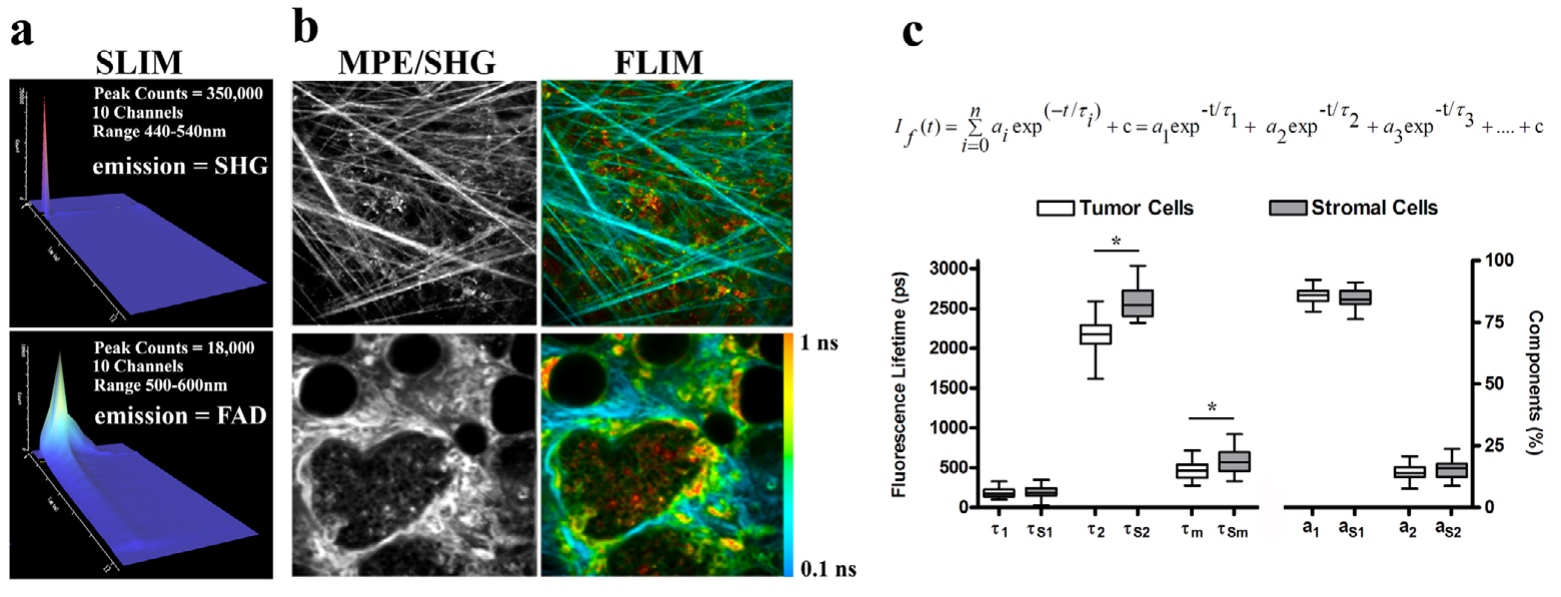
**FLIM an SLIM analysis of mammary tumors**. **(a) **Multiphoton spectral lifetime imaging microscopy (SLIM) analysis of the emission spectrum from endogenous fluorescence resulting from excitation at 890 nm. The emission signals were separated by 10 nm spectral steps over 16 channels (10 channels are displayed) and the photons collected in each channel used to generate fluorescence lifetime images and signals for each channel plotted with SLIM-Plotter (shown). Emission from collagen (at half of the input wavelength) showed a very strong and sharp signal with a no appreciable decay (lifetime) confirming the SHG nature of the collagen signal (top). Emission spectra of endogenous fluorescence from tumor and stromal cells showed that the only substantial emission signal is at 530 nm, indicating that the source of the autofluorescence signal is FAD, with lifetime values from the 530 channel matching values obtained with fluorescence lifetime imaging microscopy (FLIM). **(b) **Multiphoton intensity and FLIM images of the stroma near a tumor (top) and the tumor and stromal components (bottom) from wild-type tumors showing the utility of FLIM to image tumor cells, stromal cells, and extracellular matrix components. Note the increased intensity and fluorescent lifetimes of stromal cells (quantified in (c)) and the low lifetime of collagen (matching system response, that is, no actual lifetime/color mapping toward blue). The color map in (b) represents the weighted average of the two-term model components (τ_m _= (*a*_1_τ_1 _+ *a*_2_τ_2_)/(*a*_1 _+ *a*_2_)) using the equation shown in (c). **(c) **Quantitative analysis of fluorescent lifetime components from tumor and stromal (subscript s) cells using the equation shown. Note the increase in the second (long) component and weighted mean component (see the equation above) for stromal cells when compared with cells from the primary tumor mass. Note that at least 30 measurements per tumor image from 4 independent tumors were used to calculate lifetime values for tumor cells in the primary tumor mass while at least 6 measurements per tumor image from 4 independent tumors were used for stromal cells. *Indicates a statistically significant (*p *< 0.05) difference following analysis with one-way analysis of variance (ANOVA) with a *post-hoc *Tukey-Kramer test.

In concert with changes in the alignment of stromal collagen and increased local invasion, higher cellular autofluorescence intensity was observed in stromal cells and invading tumor cells when compared with cells in the primary tumor mass (Figures 5 and 6). To examine these progression-associated changes in more detail, we imaged the tumors with multiphoton FLIM and SLIM [32,33,39]. Using SLIM, the peak cellular emission was detected at 530 nm. Hence, the spectral properties, or 'fingerprint', of the endogenous cellular fluorophore identified it as flavin adenine dinucleotide (FAD), and not nicotinamide adenine dinucleotide (NADH) or tryptophan [40], and confirmed the presence of collagen (Figure 6a and Additional file 1), which has a theoretical zero fluorescence lifetime that is experimentally equal to the system signal response due to background noise (that is, 100 ns (blue) in Figure 6b).

Exploiting cellular FAD as an endogenous biomarker to visualize cells, we further explored the difference in FAD signal between stromal and tumor cells, using FLIM. Differences in the fluorescence lifetime of FAD between primary tumor cells and stromal cells were color mapped (Figure 6b) and quantified (Figure 6c). Stromal cells possessed a higher second component (τ_2_) and weighted mean (τ_m_) of the fluorescent lifetime, allowing stromal cells to be easily differentiated from epithelial tumor cells (Figure 6b and 6c).

**Figure 7:**
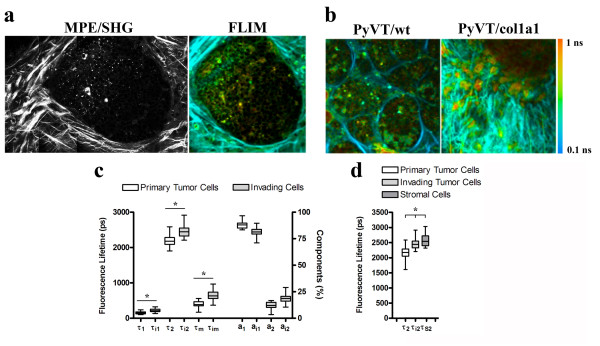
**Fluorescence lifetime imaging microscopy analysis of invading tumor cells**. **(a) **Intensity and fluorescence lifetime imaging microscopy (FLIM) images of cells away from and near invasive TACS-3 regions showing increased fluorescent intensity and lifetime near invasive regions (left side of images). **(b) **FLIM images of tumors from 10-week-old PyVT/wt and PyVT/Col1a1 animals confirming the increased TACS-3 for collagen-dense tumors shown in Figure 5. **(c) **Increased fluorescent lifetimes for invading cells. Like stromal cells the second (long) and mean components are increased in invading cells. However, the short component is also increased in invading cells when compared to cells in the primary tumor mass. Note, 45 measurements for cells within the primary tumor mass and 45 measurements for invading cells adjacent to the tumor primary tumor mass were used to calculate lifetime values. **(d) **The second (long) component from cells within the primary tumor mass, invading tumor cells, and stromal cells showing a progressive increase as cells move from a primary epithelial tumor phenotype to a more migratory phenotype. *Indicates a statistically significant (*p *< 0.05) difference following analysis with one-way analysis of variance (ANOVA) with a *post-hoc *Tukey-Kramer test.

Interestingly, invading cells displayed a fluorescent intensity more closely resembling stromal cells than cells from the primary tumor mass (Figure 7a and 7b). Consistent with this finding, changes in fluorescent intensity and fluorescent lifetimes of NADH and tryptophan have also been associated with cells of differing metastatic potential [41]. Because invading tumor cells commonly de-differentiate, it is possible that shifts in the fluorescent lifetime may be indicative of fundamental changes in cells associated with invasion and metastasis. In fact, a metabolic signature of higher FAD fluorescent intensity was observed in cells near invading regions when compared with non-invading regions (Figure 7a) while invading tumor cells showed a longer FAD fluorescent lifetime (the right panel in Figure 7b), having higher first (τ_1_), second (τ_2_), and weighted mean (τ_m_) lifetime components (Figure 7c), and could be differentiated from stromal cells and cells in the primary tumor mass. Furthermore, examination of τ_2 _values indicates a progressive increase in lifetimes from cells within the tumor mass to invading cells to stromal cells (Figure 7d) supporting the idea of a fundamental change to invading cancer cells.

In addition to identifying key differences in measurable fluorescent intensity and lifetime associated with invading cells, FLIM analysis confirmed results shown in Figure 4 demonstrating a shift towards TACS-3 and increased local invasion with higher collagen density (see Figure 7b). Invading cells associated with TACS-3 could be clearly differentiated in collagen-dense tissues (the right panel in Figure 7b) while PyVT/wt tumors (the left panel in Figure 7b) were not highly invasive at this stage (week 10).

## Discussion

### Collagen density and tumor formation and progression

Although the increased risk for breast carcinoma associated with collagen-dense breast tissue has been described [1-3], a causal link between increased stromal collagen and increased breast carcinoma has not been previously established. Moreover, little is known about the molecular mechanisms underlying increased collagen deposition and its influence on the interactions between stromal collagen, fibroblasts, and epithelial cells, or how increased collagen affects tumorigenesis and tumor cell phenotype. This is due in large part to the fact that no animal model system had previously existed to study these phenomena *in vivo*. Herein we demonstrate that mice with increased stromal collagen have increased mammary tumors that are more invasive and metastatic, and thus provide a causal link between stromal density and carcinoma progression, consistent with reports of human breast carcinoma risk.

**Figure 8:**
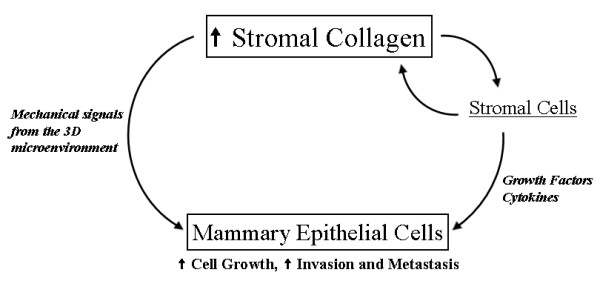
**Model for advancement of mammary epithelial tumors by increased stromal collagen**. Increased fibrillar collagen in the mammary stroma directly regulates the three-dimensional mechanical microenvironment of mammary epithelial cells, influencing proliferation and phenotype. In addition, increased collagen advances a feed-forward loop with fibroblasts to promote additional collagen deposition and an increased stromal/fibroblast population resulting in increased paracrine signaling to mammary epithelial cells. The net result is increased epithelial proliferation/tumor formation and a more invasive and metastatic phenotype.

In this system, increased collagen density is the initial event, promoting tumor initiation and metastasis. This may be the result of two likely mechanisms (Figure 8), both of which follow the increase in collagen density (that is, increasing collagen is the initial event in our system). The first mechanism is that increased breast density is associated with a stiffer extracellular matrix resulting in high local mechanical loads and higher resistance to cellular contractility for breast epithelial cells. Such changes in the physical microenvironment has been shown to alter focal adhesion and Rho GTPase signaling, resulting in increased proliferation and a more transformed phenotype [24,25]. A second, and more indirect mechanism, may be the influence of increased stromal collagen on mammary fibroblasts that in turn influence epithelial cells. Stromal fibroblasts can regulate epithelial cells in part through secretion of specific soluble growth factors and chemokines [20,42-44]. For instance, TGF-β has been associated with reactive stroma, fibrosis, and epithelial cell invasion [45], while numerous studies indicate that the epidermal growth factor (for example, EGFR, HER-2/neu/ErbB2, ErbB3, and so on), insulin-like growth factor (for example, IGF-I, IGFBP3, and so on), and hepatocyte growth/scatter factor (HGF/SF, c-Met) systems are important not only in the normal mammary gland but also during tumorigenesis and metastasis [44,46-49]. Furthermore, the IGF family has been implicated in association with dense breast tissue [14,50,51] with both local [14] and circulating [50,51] levels of IGF-I positively correlated with breast tissue density. In fact, both of these mechanisms are plausible and are likely to be acting in concert with one another to produce fundamental changes in both the breast epithelial and stromal cells. Since both adhesion-mediated and growth factor-mediated signaling pathways are often interrelated [52-57], understanding each of these possible mechanisms and their convergence is likely to be of great importance to understanding breast tissue density-related carcinoma.

However, the possibility that altered matrix remodeling associated with the Col1a1 model is playing a role also warrants consideration. However, in theory, a significant defect in matrix remodeling should inhibit tumor progression, and the fraction of collagen that is collagenase-resistant can be degraded/remodeled at a second site by the rodent collagenase and other proteases that are expressed by tumor and tumor-associated cells. Hence, while limitations of the model must be taken into account when considering the presented data, it appears unlikely that a defect in matrix remodeling associated with the use of the Col1a1 model is causal for the increases in tumor formation and progression observed in this study.

### Collagen signatures and local invasion

In a previous study we described the use of collagen alignment to quantify local invasion with the level of TACS-2 (alignment tangential to the tumor boundary at a 0° angle) and TACS-3 (alignment radial to the tumor boundary at an angle of 90°) providing a novel quantitative assessment of tumor progression [27]. In this study, the analysis of collagen radial alignment in samples from 8- and 10-week tumors demonstrates a transition from TACS-2 to TACS-3. We observe a broad distribution of fiber angles away from zero but not yet tightly grouped as late-stage tumor at the radial alignment (90°) associated with the high degree of local invasion previously reported for 15-week tumors [27]. This result suggests that the move toward invasive behavior is a transitional process increasing with time. We find that tumor cells in collagen-dense tumors are not only more invasive and metastatic *in vivo*, but were also more invasive and migratory *in vitro *(Figure 3b and 3c), indicating that the increased invasiveness is not only the result of earlier tumorigenesis that had more time to progress, but also due to tumor cells that are fundamentally more invasive because they arose within collagen-dense tissues. This finding suggests that cellular behavior is altered by epigenetic changes signaled from the collagen-dense stroma, consistent with findings that increased collagen density alters epithelial cell signaling and behavior *in vitro *[25].

### FAD and local invasion

Interestingly, we measured an increase in the fluorescence lifetime for the metabolite FAD associated with invading cells. While this information provides a valuable biomarker for use with an optical biopsy, the biological relevance of this finding is not well understood. It is known that transformed cells often undergo increased glycolysis in the cytosol, a phenomena known as the Warburg effect [58], and that the fluorescent lifetimes of NADH and FAD, and in particular the redox ratio of these two metabolites, is altered in transformed cells [59]. Of interest, Skala and co-workers [59] recently reported an increase in the τ_1 _component of the FAD lifetime in precancerous cells when compared with normal epithelium. In the current study we compare non-invasive transformed cells with invading cells, and as such we speculate that the alteration in FAD state seen in transformation may become increasingly mis-regulated in the more metastatic population of transformed cells. Furthermore, the biological implications of the increased FAD intensity and fluorescent lifetime may be found in the possibility that increased glycolysis is increasing levels of NADH, a known regulator of transcription [60], and resulting in more lactic acid production [61] with less pyruvate entering into the citric acid cycle and, as a consequence, less FAD being reduced to FADH_2_. Moreover, it has also been reported that the fluorescence lifetime of FAD can decrease due to quenching from NAD+, other molecular interactions, or environmental conditions [62,63], and thus the increased fluorescence lifetime of FAD could also be indicative of less available NAD+, particularly in the cytosol, and other unknown changes in FAD binding and localization. Hence, the biological implications of altered FAD intensity and fluorescence lifetime remain elusive. However, our results provide evidence that changes in FAD signals can be found within a more invasive subpopulation of carcinoma cells and as such understanding the regulatory mechanisms associated with these observations may provide great insight into tumor cell metastasis.

## Conclusion

In summary, increased collagen density increases tumorigenesis, local invasion, and metastasis, causally linking increased stromal collagen to tumor formation and progression. Imaging with combined MPE and SHG in tumors allows visualization of cellular autofluorescence and defined collagen structures that identify key differences associated with high collagen density and may provide useful diagnostic tools to rapidly assess fresh tissue biopsies. Furthermore, imaging live tissues with FLIM and SLIM confirms results obtained with MPE/SHG and identifies significant differences in fluorescence lifetimes that are indicative of invasive cells. Thus, FLIM and SLIM serve as powerful tools to evaluate the invasiveness of tumor cells in mammary tissues. Given the significant findings associated with high breast tissue density and the now available utility of a mouse model for breast tissue density, fundamental questions regarding the molecular mechanisms associated with breast tissue density-related carcinoma can now be further addressed *in vivo*.

## List of abbreviations

ANOVA: analysis of variance; BSA, bovine serum albumin; DCIS: ductal carcinoma *in situ*; DMEM: Dulbecco/Vogt modified Eagle's minimal essential medium; H&E: hematoxylin and eosin; FAD: flavin adenine dinucleotide; FBS: fetal bovine serum; FITC: fluorescein isothiocyanate; FLIM: fluorescence lifetime imaging microscopy; MMP: matrix metalloproteinase; MPLSM: multiphoton laser-scanning microscopy; MPE: multiphoton excitation; NA: numerical aperture; NADH: nicotinamide adenine dinucleotide; PBS: phosphate buffered saline; PCR: polymerase chain reaction; PMT: photomultiplier tube; PyVT: polyomavirus middle-T; SHG: second harmonic generation; SLIM: spectral-lifetime imaging microscopy; TACS: tumor-associated collagen signature; TCSPC: time-correlated single photon counting; TRITC: tetramethylrhodamine isothiocyanate.

## Competing interests

The authors declare that they have no competing interests. Portions of the technologies presented in the manuscript are patented or patent pending. However, the authors have no interest, arrangement, or affiliation that could be perceived as a conflict of interest in the context of this manuscript.

## Authors' contributions

PPP conducted all MPLSM, SHG, FLIM, and histology experiments, managed the mouse colonies and tumor studies, performed three-dimensional cell culture experiments, analyzed the imaging data, and prepared the manuscript and figures. DRI and JGK assisted with mice and tumor studies, and performed three-dimensional culture experiments. KWE and JGW assisted with specific technical aspects of nonlinear imaging and data analysis as well as project coordination. LY conducted SLIM imaging with PPP and assisted in data analysis. CTR contributed to the analysis of imaging data and the development of computational tools for SLIM analysis. PJK participated in the design and coordination of the project and assisted with data analysis. PPP, KWE, JGW, and PJK cooperatively designed the project and discussed data interpretation and analysis. All authors participated in critical editing of the manuscript.

## Pre-publication history

The pre-publication history for this paper can be accessed here:



## Supplementary Material

Additional file 1 The multiphoton spectral lifetime imaging microscopy (SLIM) analysis of live tumors. Multiphoton fluorescence lifetime imaging microscopy (FLIM) demonstrates the measurable fluorescence lifetimes of live tumor cells as shown in Figures 6 and 7. Using SLIM, the fluorescence lifetimes following 890 nm two-photon excitation of live three-dimensional tumors are measured within a defined spectra, allowing identification of the emitting fluorophore and noise removal from adjacent spectra. For instance, examination of the 440–450 nm emission spectra from SLIM confirms the presence of collagen bounding tumor cells. For an 890 nm two-photon excitation the second harmonic generation (SHG) signal is maximal at 445 nm and has no lifetime (dark blue). In addition, the maximal emission signal from tumor cells is 535 nm as shown in Figure 6a, indicating the emission results from excitation of the endogenous fluorophore flavin adenine dinucleotide (FAD). Color bar 0 to 1 ns. Click here for file

## References

[B1] McCormack VA, dos Santos Silva I (2006). Breast density and parenchymal patterns as markers of breast cancer risk: a meta-analysis. Cancer Epidemiol Biomarkers Prev.

[B2] Boyd NF, Lockwood GA, Byng JW, Tritchler DL, Yaffe MJ (1998). Mammographic densities and breast cancer risk. Cancer Epidemiol Biomarkers Prev.

[B3] Boyd NF, Martin LJ, Stone J, Greenberg C, Minkin S, Yaffe MJ (2001). Mammographic densities as a marker of human breast cancer risk and their use in chemoprevention. Curr Oncol Rep.

[B4] Boyd NF, Dite GS, Stone J, Gunasekara A, English DR, McCredie MR, Giles GG, Tritchler D, Chiarelli A, Yaffe MJ, Hopper JL (2002). Heritability of mammographic density, a risk factor for breast cancer. N Engl J Med.

[B5] Boyd NF, Rommens JM, Vogt K, Lee V, Hopper JL, Yaffe MJ, Paterson AD (2005). Mammographic breast density as an intermediate phenotype for breast cancer. Lancet Oncol.

[B6] Rutter CM, Mandelson MT, Laya MB, Seger DJ, Taplin S (2001). Changes in breast density associated with initiation, discontinuation, and continuing use of hormone replacement therapy. JAMA.

[B7] Ursin G, Hovanessian-Larsen L, Parisky YR, Pike MC, Wu AH (2005). Greatly increased occurrence of breast cancers in areas of mammographically dense tissue. Breast Cancer Res.

[B8] Alowami S, Troup S, Al-Haddad S, Kirkpatrick I, Watson PH (2003). Mammographic density is related to stroma and stromal proteoglycan expression. Breast Cancer Res.

[B9] Gill JK, Maskarinec G, Pagano I, Kolonel LN (2006). The association of mammographic density with ductal carcinoma *in situ *of the breast: the Multiethnic Cohort. Breast Cancer Res.

[B10] Habel LA, Dignam JJ, Land SR, Salane M, Capra AM, Julian TB (2004). Mammographic density and breast cancer after ductal carcinoma in situ. J Natl Cancer Inst.

[B11] Aiello EJ, Buist DS, White E, Porter PL (2005). Association between mammographic breast density and breast cancer tumor characteristics. Cancer Epidemiol Biomarkers Prev.

[B12] Hawes D, Downey S, Pearce CL, Bartow S, Wan P, Pike MC, Wu AH (2006). Dense breast stromal tissue shows greatly increased concentration of breast epithelium but no increase in its proliferative activity. Breast Cancer Res.

[B13] Li T, Sun L, Miller N, Nicklee T, Woo J, Hulse-Smith L, Tsao MS, Khokha R, Martin L, Boyd N (2005). The association of measured breast tissue characteristics with mammographic density and other risk factors for breast cancer. Cancer Epidemiol Biomarkers Prev.

[B14] Guo YP, Martin LJ, Hanna W, Banerjee D, Miller N, Fishell E, Khokha R, Boyd NF (2001). Growth factors and stromal matrix proteins associated with mammographic densities. Cancer Epidemiol Biomarkers Prev.

[B15] Barcellos-Hoff MH, Aggeler J, Ram TG, Bissell MJ (1989). Functional differentiation and alveolar morphogenesis of primary mammary cultures on reconstituted basement membrane. Development.

[B16] Keely P, Fong A, Zutter M, Santoro S (1995). Alteration of collagen-dependent adhesion, motility, and morphogenesis by the expression of antisense α2 integrin mRNA in mammary cells. J Cell Sci.

[B17] Tlsty TD, Hein PW (2001). Know thy neighbor: stromal cells can contribute oncogenic signals. Curr Opin Genet Dev.

[B18] Noel A, Foidart JM (1998). The role of stroma in breast carcinoma growth *in vivo *. J Mammary Gland Biol Neoplasia.

[B19] Elenbaas B, Spirio L, Koerner F, Fleming MD, Zimonjic DB, Donaher JL, Popescu NC, Hahn WC, Weinberg RA (2001). Human breast cancer cells generated by oncogenic transformation of primary mammary epithelial cells. Genes Dev.

[B20] Orimo A, Gupta PB, Sgroi DC, Arenzana-Seisdedos F, Delaunay T, Naeem R, Carey VJ, Richardson AL, Weinberg RA (2005). Stromal fibroblasts present in invasive human breast carcinomas promote tumor growth and angiogenesis through elevated SDF-1/CXCL12 secretion. Cell.

[B21] Shekhar MP, Pauley R, Heppner G, Werdell J, Santner SJ, Pauley RJ, Tait L (2003). Host microenvironment in breast cancer development: extracellular matrix-stromal cell contribution to neoplastic phenotype of epithelial cells in the breast. Breast Cancer Res.

[B22] Iyengar P, Espina V, Williams TW, Lin Y, Berry D, Jelicks LA, Lee H, Temple K, Graves R, Pollard J (2005). Adipocyte-derived collagen VI affects early mammary tumor progression *in vivo*, demonstrating a critical interaction in the tumor/stroma microenvironment. J Clin Invest.

[B23] White DE, Kurpios NA, Zuo D, Hassell JA, Blaess S, Mueller U, Muller WJ (2004). Targeted disruption of beta1-integrin in a transgenic mouse model of human breast cancer reveals an essential role in mammary tumor induction. Cancer Cell.

[B24] Paszek MJ, Zahir N, Johnson KR, Lakins JN, Rozenberg GI, Gefen A, Reinhart-King CA, Margulies SS, Dembo M, Boettiger D (2005). Tensional homeostasis and the malignant phenotype. Cancer Cell.

[B25] Wozniak MA, Desai R, Solski PA, Der CJ, Keely PJ (2003). ROCK-generated contractility regulates breast epithelial cell differentiation in response to the physical properties of a three-dimensional collagen matrix. J Cell Biol.

[B26] Liu X, Wu H, Byrne M, Jeffrey J, Krane S, Jaenisch R (1995). A targeted mutation at the known collagenase cleavage site in mouse type I collagen impairs tissue remodeling. J Cell Biol.

[B27] Provenzano PP, Eliceiri KW, Campbell JM, Inman DR, White JG, Keely PJ (2006). Collagen reorganization at the tumor-stromal interface facilitates local invasion. BMC Medicine.

[B28] Williams RM, Zipfel WR, Webb WW (2005). Interpreting second-harmonic generation images of collagen I fibrils. Biophys J.

[B29] Nazir MZ, Eliceiri KW, Ahmed A, Hathaway E, Hashmi A, Agarwal V, Rao Y, Kumar S, Lukas T, Riching KM, Rueden C, Wang Y, White JG (2006). WiscScan: a software defined laser-scanning microscope. Biomed Eng Online.

[B30] Rueden C, Eliceiri KW, White JG (2004). VisBio: a computational tool for visualization of multidimensional biological image data. Traffic.

[B31] ImageJ. http://rsb.info.nih.gov/ij/.

[B32] Bird DK, Eliceiri KW, Fan CH, White JG (2004). Simultaneous two-photon spectral and lifetime fluorescence microscopy. Appl Opt.

[B33] Provenzano PP, Rueden CT, Trier SM, Yan L, Ponik SM, Inman DR, Keely PJ, Eliceiri KW Nonlinear optical imaging and spectral-lifetime computational analysis of endogenous and exogenous fluorophores in breast cancer. J Biomed Opt.

[B34] Lin EY, Jones JG, Li P, Zhu L, Whitney KD, Muller WJ, Pollard JW (2003). Progression to malignancy in the polyoma middle T oncoprotein mouse breast cancer model provides a reliable model for human diseases. Am J Pathol.

[B35] Wang W, Wyckoff JB, Frohlich VC, Oleynikov Y, Huttelmaier S, Zavadil J, Cermak L, Bottinger EP, Singer RH, White JG (2002). Single cell behavior in metastatic primary mammary tumors correlated with gene expression patterns revealed by molecular profiling. Cancer Res.

[B36] Zipfel WR, Williams RM, Christie R, Nikitin AY, Hyman BT, Webb WW (2003). Live tissue intrinsic emission microscopy using multiphoton-excited native fluorescence and second harmonic generation. Proc Natl Acad Sci USA.

[B37] Zoumi A, Yeh A, Tromberg BJ (2002). Imaging cells and extracellular matrix *in vivo *by using second-harmonic generation and two-photon excited fluorescence. Proc Natl Acad Sci USA.

[B38] Brown E, McKee T, diTomaso E, Pluen A, Seed B, Boucher Y, Jain RK (2003). Dynamic imaging of collagen and its modulation in tumors *in vivo *using second-harmonic generation. Nat Med.

[B39] Yan L, Rueden CT, White JG, Eliceiri KW (2006). Applications of combined spectral lifetime microscopy for biology. Biotechniques.

[B40] Huang S, Heikal AA, Webb WW (2002). Two-photon fluorescence spectroscopy and microscopy of NAD(P)H and flavoprotein. Biophys J.

[B41] Pradhan A, Pal P, Durocher G, Villeneuve L, Balassy A, Babai F, Gaboury L, Blanchard L (1995). Steady state and time-resolved fluorescence properties of metastatic and non-metastatic malignant cells from different species. J Photochem Photobiol B.

[B42] Bavik C, Coleman I, Dean JP, Knudsen B, Plymate S, Nelson PS (2006). The gene expression program of prostate fibroblast senescence modulates neoplastic epithelial cell proliferation through paracrine mechanisms. Cancer Res.

[B43] Allinen M, Beroukhim R, Cai L, Brennan C, Lahti-Domenici J, Huang H, Porter D, Hu M, Chin L, Richardson A (2004). Molecular characterization of the tumor microenvironment in breast cancer. Cancer Cell.

[B44] Chung LW, Baseman A, Assikis V, Zhau HE (2005). Molecular insights into prostate cancer progression: the missing link of tumor microenvironment. J Urol.

[B45] De Wever O, Mareel M (2003). Role of tissue stroma in cancer cell invasion. J Pathol.

[B46] Condeelis J, Singer RH, Segall JE (2005). The great escape: when cancer cells hijack the genes for chemotaxis and motility. Annu Rev Cell Dev Biol.

[B47] Parr C, Watkins G, Mansel RE, Jiang WG (2004). The hepatocyte growth factor regulatory factors in human breast cancer. Clin Cancer Res.

[B48] Sachdev D, Yee D (2001). The IGF system and breast cancer. Endocr Relat Cancer.

[B49] Surmacz E (2000). Function of the IGF-I receptor in breast cancer. J Mammary Gland Biol Neoplasia.

[B50] Byrne C, Colditz GA, Willett WC, Speizer FE, Pollak M, Hankinson SE (2000). Plasma insulin-like growth factor (IGF) I, IGF-binding protein 3, and mammographic density. Cancer Res.

[B51] Boyd NF, Stone J, Martin LJ, Jong R, Fishell E, Yaffe M, Hammond G, Minkin S (2002). The association of breast mitogens with mammographic densities. Br J Cancer.

[B52] Benlimame N, He Q, Jie S, Xiao D, Xu YJ, Loignon M, Schlaepfer DD, Alaoui-Jamali MA (2005). FAK signaling is critical for ErbB-2/ErbB-3 receptor cooperation for oncogenic transformation and invasion. J Cell Biol.

[B53] Aplin AE, Juliano RL (1999). Integrin and cytoskeletal regulation of growth factor signaling to the MAP kinase pathway. J Cell Sci.

[B54] Baron V, Calleja V, Ferrari P, Alengrin F, Van Obberghen E (1998). p125Fak focal adhesion kinase is a substrate for the insulin and insulin-like growth factor-I tyrosine kinase receptors. J Biol Chem.

[B55] Ishizawar R, Parsons SJ (2004). c-Src and cooperating partners in human cancer. Cancer Cell.

[B56] Hauck CR, Sieg DJ, Hsia DA, Loftus JC, Gaarde WA, Monia BP, Schlaepfer DD (2001). Inhibition of focal adhesion kinase expression or activity disrupts epidermal growth factor-stimulated signaling promoting the migration of invasive human carcinoma cells. Cancer Res.

[B57] Sieg DJ, Hauck CR, Ilic D, Klingbeil CK, Schaefer E, Damsky CH, Schlaepfer DD (2000). FAK integrates growth-factor and integrin signals to promote cell migration. Nat Cell Biol.

[B58] Warburg O (1930). The Metabolism of Tumors.

[B59] Skala MC, Riching KM, Gendron-Fitzpatrick A, Eickhoff J, Eliceiri KW, White JG, Ramanujam N (2007). *In vivo *multiphoton microscopy of NADH and FAD redox states, fluorescence lifetimes, and cellular morphology in precancerous epithelia. Proc Natl Acad Sci USA.

[B60] Garriga-Canut M, Schoenike B, Qazi R, Bergendahl K, Daley TJ, Pfender RM, Morrison JF, Ockuly J, Stafstrom C, Sutula T (2006). 2-Deoxy-D-glucose reduces epilepsy progression by NRSF-CtBP-dependent metabolic regulation of chromatin structure. Nat Neurosci.

[B61] Gatenby RA, Gawlinski ET, Gmitro AF, Kaylor B, Gillies RJ (2006). Acid-mediated tumor invasion: a multidisciplinary study. Cancer Res.

[B62] Lakowicz JR (2006). Principles of Fluorescence Spectroscopy.

[B63] Maeda-Yorita K, Aki K (1984). Effect of nicotinamide adenine dinucleotide on the oxidation-reduction potentials of lipoamide dehydrogenase from pig heart. J Biochem.

