# Supplementary figures and images for: Collagen density promotes mammary tumor initiation and progression
Source: BMC Med. 2008 Apr 28;6:11. doi: 10.1186/1741-7015-6-11 (PMC2386807; doi:10.1186/1741-7015-6-11)

2P  $\lambda_{\text{ex}} = 890\text{nm}$

FLIM

SLIM

Intensity  
(MPE/SHG)

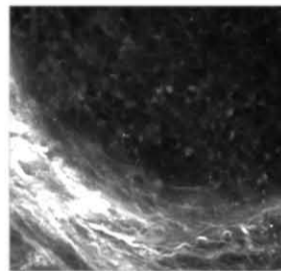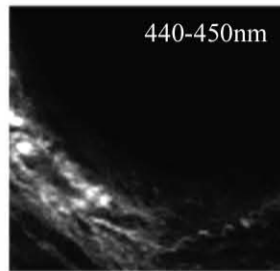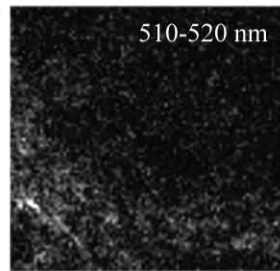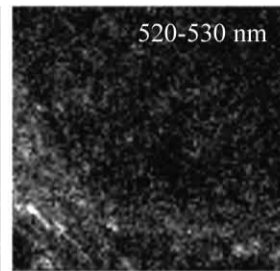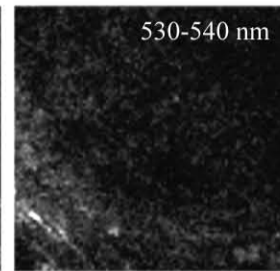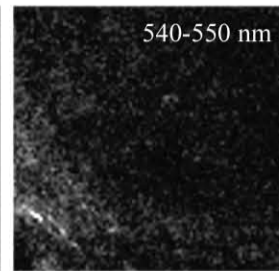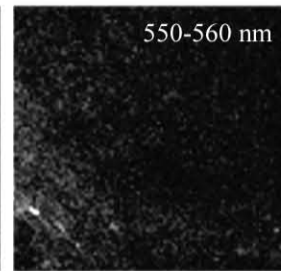

Mean  
Fluorescence  
Lifetime

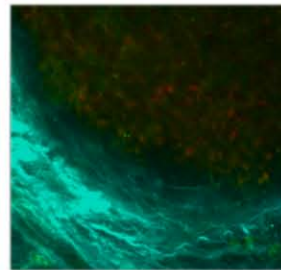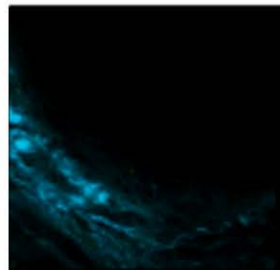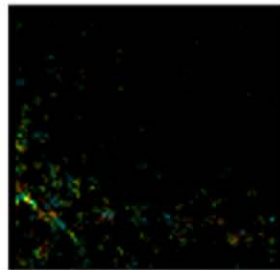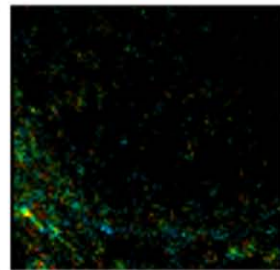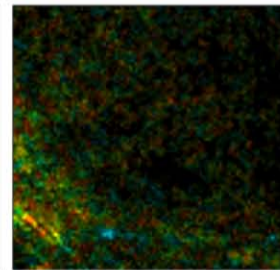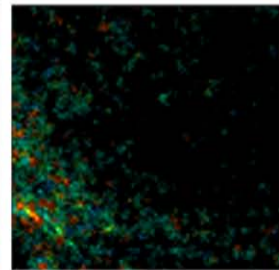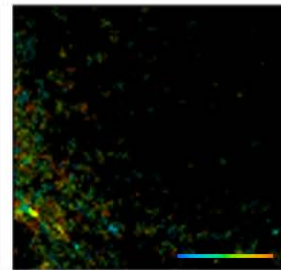

Supplement: Additional file 1 — The multiphoton spectral lifetime imaging microscopy (SLIM) analysis of live tumors. Multiphoton fluorescence lifetime imaging microscopy (FLIM) demonstrates the measurable fluorescence lifetimes of live tumor cells as shown in Figures 6 and 7. Using SLIM, the fluorescence lifetimes following 890 nm two-photon excitation of live three-dimensional tumors are measured within a defined spectra, allowing identification of the emitting fluorophore and noise removal from adjacent spectra. For instance, examination of the 440–450 nm emission spectra from SLIM confirms the presence of collagen bounding tumor cells. For an 890 nm two-photon excitation the second harmonic generation (SHG) signal is maximal at 445 nm and has no lifetime (dark blue). In addition, the maximal emission signal from tumor cells is 535 nm as shown in Figure 6a, indicating the emission results from excitation of the endogenous fluorophore flavin adenine dinucleotide (FAD). Color bar 0 to 1 ns. [file 1741-7015-6-11-S1.pdf]
